# A Novel LiDAR-Based Instrument for High-Throughput, 3D Measurement of Morphological Traits in Maize and Sorghum

**DOI:** 10.3390/s18041187

**Published:** 2018-04-13

**Authors:** Suresh Thapa, Feiyu Zhu, Harkamal Walia, Hongfeng Yu, Yufeng Ge

**Affiliations:** 1 Department of Biological Systems Engineering, University of Nebraska-Lincoln, Lincoln, NE 68583, USA; Suresh.thapa@huskers.unl.edu; 2 Department of Computer Science and Engineering, University of Nebraska-Lincoln, Lincoln, NE 68588, USA; Feiyuzhu520@gmail.com (F.Z.); hfyu@unl.edu (H.Y.); 3 Department of Agronomy and Horticulture, University of Nebraska-Lincoln, Lincoln, NE 68583, USA; hwalia2@unl.edu

**Keywords:** high-throughput plant phenotyping, leaf area, leaf inclination angle, leaf angular distribution, 3D point cloud, LiDAR

## Abstract

Recently, imaged-based approaches have developed rapidly for high-throughput plant phenotyping (HTPP). Imaging reduces a 3D plant into 2D images, which makes the retrieval of plant morphological traits challenging. We developed a novel LiDAR-based phenotyping instrument to generate 3D point clouds of single plants. The instrument combined a LiDAR scanner with a precision rotation stage on which an individual plant was placed. A LabVIEW program was developed to control the scanning and rotation motion, synchronize the measurements from both devices, and capture a 360° view point cloud. A data processing pipeline was developed for noise removal, voxelization, triangulation, and plant leaf surface reconstruction. Once the leaf digital surfaces were reconstructed, plant morphological traits, including individual and total leaf area, leaf inclination angle, and leaf angular distribution, were derived. The system was tested with maize and sorghum plants. The results showed that leaf area measurements by the instrument were highly correlated with the reference methods (R^2^ > 0.91 for individual leaf area; R^2^ > 0.95 for total leaf area of each plant). Leaf angular distributions of the two species were also derived. This instrument could fill a critical technological gap for indoor HTPP of plant morphological traits in 3D.

## 1. Introduction

Image-based approaches to rapidly and nondestructively measure plant morphological traits have emerged and quickly developed in response to the need for accelerating high-throughput plant phenotyping that would eventually enable effective use of genomic data to bridge the genotype-to-phenotype gap for crop improvement [1,2]. Image analysis has been used successfully to measure the physical traits of plant shoots including height, leaf area, and biomass [3,4,5]. More importantly, by combining information from a series of plant images over time, it has become feasible to capture dynamic traits, such as growth rate, rate of senescence, and estimation of plant architecture. The approaches that involve quantifiable dynamic traits have the potential to significantly impact targeted crop research for increased stress tolerance as stress responses in plants are highly dynamic.

Accurate measurement of the 3D structure of a plant is important for the study of plant phenomics. The 3D structure (shape, size, angle, number, etc.) of leaves affects the physiological processes of the plant. For example, plant leaf area and angle significantly influence light interception and apparent transpiration, photosynthesis, and plant productivity [6]. Leaf area must be considered along with the leaf angle to derive their net impact on the photosynthetic efficiency of the plant. Further, leaf angle is an important determinant of optimal planting density in many crops. Field investigations of the relationships between leaf angle and grain yield and apparent photosynthesis suggested higher grain yields for the hybrid with erect leaves than its counterpart with horizontal type leaves [7]. The spatial distribution of radiation and light interception can be derived from the 3D plant architecture, combined with environmental factors and physiological leaf properties [8].

Traditional techniques available for measuring leaf area are laborious, time consuming and usually destructive. One of the earliest methods is to trace the outline of the leaves on a paper and calculate the area from it [9]. Some other procedures include allometric models and equations to convert the leaf length and width to the leaf area [10]. Similarly, manual measurement of leaf angle involves the procedure of aligning an inclinometer with each leaf segment on a plant [11]. Obviously, these methods are not suitable to measure hundreds of plants continuously in a modern plant phenotyping setting that requires rapid and nondestructive assays.

Although image-based approaches have been applied to measure plant structures in 3D, it has intrinsic limitations. Because imaging projects a 3D object onto a 2D image plane, the depth information is lost. This in turn causes an occlusion problem, where part of the plant is not visible in a specific image. To infer the total leaf area or biomass from the projected leaf areas, empirical relationships are usually developed from multi-view images (usually two to three side views and one top view, [12]). However, such relationships are usually species dependent and may not be applied broadly to a range of plant species or growth stages.

To overcome these drawbacks, stereo imaging (and similarly, Structure-From-Motion, SFM) is brought to generate 3D point clouds of plants by capturing two images of a scene from two cameras (or one camera at two positions in SFM) [13]. The method was tested in plant phenotyping, with applications in soybean [14], wheat and rice [15], and rape [16]. The accuracy of stereo imaging/SFM depends on the ability to match the corresponding points in the image pair to gain the depth information, which can be sensitive to environmental conditions, ineffective for low texture scenes, and computationally intensive for large images [17]. 

LiDAR (Light Detection and Ranging) employs the time-of-flight principle of active lasers to acquire 3D information of an object. LiDAR was originally developed for terrain, benthic and forest surveys from aerial platforms [18]. Later on terrestrial LiDAR scanning (TLS) systems were developed and used to measure the 3D structural properties of buildings, bridges, and trees. More recently, the applications of TLS systems in agriculture have also been reported. For example, Eitel et al. [19] demonstrated the feasibility of a green TLS to measure dry biomass and the nitrogen concentration of winter wheat crops. Friedli et al. [20] showed that a 3D TLS allowed for the measurement of canopy height growth of maize, soybean, and wheat with high temporal resolution. More recently, LiDAR sensors were mounted on mobile platforms to measure height and above ground biomass of field grown crops [21,22,23].

Because of its capability to measure structure and shape in 3D and because of the decrease in its price with the advancement in technology, LiDAR is regarded as an important technology to complement 2D image analysis for high-throughput plant phenotyping [24]. It would be particularly useful to measure the structural and morphological properties of plant shoots such as leaf area and leaf orientation. These parameters are most accurately quantified in 3D space, and it is a challenging task to reconstruct them from a set of 2D images. Paulus et al. [25] used a precision laser scanning device (a LiDAR based on continuous wave phase modulation) coupled to an articulated measuring arm to generate 3D point clouds of barley (*Hordeum vulgare* L.) plants. They showed that traits, including leaf area and stem height, could be quantified through the processing of LiDAR point clouds. This study clearly demonstrated the usefulness of LiDAR to measure the structural parameters of single plants. However, the use of an articulated arm to direct the laser beam onto the plant surface involved a manual process. To measure larger plants, such as maize or sorghum, the measurement time would also be significantly longer. Because of the strong need to link genotype to phenotype, automated and faster approaches are needed to increase the throughput and flexibility of a LiDAR based system.

In this paper, we report the development of a novel LiDAR-based sensing system that can automatically produce 3D point clouds of plants with a 360-degree view. We have developed a fully integrated data processing pipeline to reconstruct plant leaf surfaces and derive leaf area, leaf inclination angle, and leaf angular distribution. Finally, we provide performance validation for the system on two agronomically and economically important crop species: maize and sorghum.

## 2. Materials and Methods

### 2.1. Description of Hardware and Software of the Instrument

**Figure 1:**
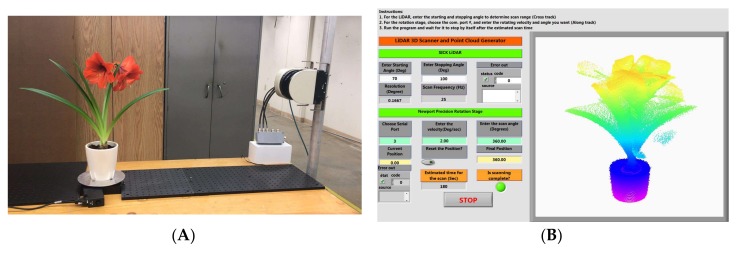
Illustration of the new instrument consisting of a LiDAR scanner and precision rotation stage for 3D measurement of plants at 360° view (**A**). The LabVIEW graphic user interface for instrument control, measurement acquisition, and real-time point cloud visualization (**B**).

The physical setup of the instrument is shown in Figure 1A. The plant is placed on a rotational stage to provide continuous rotation at 360°. A LiDAR emits laser beams that scan vertically to cover the height of the plant. The combination of the rotating and scanning motion allows for the creation of the 3D point cloud of the plant with a 360° view.

An off-the-shelf LiDAR module (LMS 511, SICK AG) was selected for instrument development. The LMS 511 operates on a time-of-flight principle to calculate the distance to a target. It uses an NIR laser diode (at 905 nm) to send out pulsed laser beams, which are then reflected from the target and received by the laser detector. Because vegetation normally has higher reflectivity in the NIR spectral range, this increases the detection and ranging accuracy of reflected laser energy. This instrument has an adjustable scanning frequency between 25 Hz and 100 Hz. At 25 Hz, the angular interval of the laser beam is 0.0029 radian. The plant is placed approximately 1.0 to 1.5 m from the LiDAR scanner. This translates to 2.9 to 4.4 mm of sampling resolution of the point cloud along the vertical scanning direction. In addition, the LiDAR has a ranging precision of 6 mm. The LiDAR connects to the computer via an Ethernet connection and data packets are transmitted using a User Datagram Protocol.

A URS 150 BCC (Newport Corporation, Irvine, CA, USA) precision rotation stage was used to provide the rotational motion of plants. It has a maximum payload capacity of 300 N. It provides continuous 360° rotation with an angular resolution of 0.002°. The stage can rotate as fast as 80°/s. In our application, the rotational speed is maintained at 3°/s, allowing 120 s for a complete rotation. Therefore, the point cloud generation time for each plant was two minutes. With the LiDAR scanning frequency at 25 Hz, the radial resolution of point clouds is 0.0021 radian. The stage connects to the computer via an RS-232 serial connection. 

A LabVIEW (National Instrument) program was developed to control and synchronize the two devices (Figure 1B). The program allows the users to set the suitable scanning parameters and visualize the raw point cloud data in real time. The swath for the LiDAR scanner is determined by the plant height, which in turn can be adjusted by entering the start angle and the stop angle of the laser beams in the program.

The URS150 BCC can be rotated in a discrete or continuous manner to acquire the entire or partial 360° degree point cloud. The continuous rotation was employed in our design, because it eliminates abrupt motions that cause plants to vibrate and lower the quality of the point clouds. Continuous rotation also improves the measurement throughput. The system also has some flexibility in determining the suitable height of the LiDAR platform and the distance to the rotation stage depending upon the size and shape of the plant. 

During the scan, after one sweep of the laser beam in the vertical direction, the data packet received from LiDAR is parsed with the LabVIEW program and written to a spreadsheet file. The data string received is encoded in hexadecimal format, which contains information about device parameters (e.g., device number, device status, time since startup, etc.), scan parameters (e.g., resolution, frequency, start angle, stop angle, etc.), distance values and reflectance values. The measured distance *R* between the LiDAR and the target point is registered along with the beam angle θ and the rotation stage angle ϕ, using the scan frequency of the LiDAR, speed of the rotation stage and the scan starting time values given by the LiDAR.

**Figure 2:**
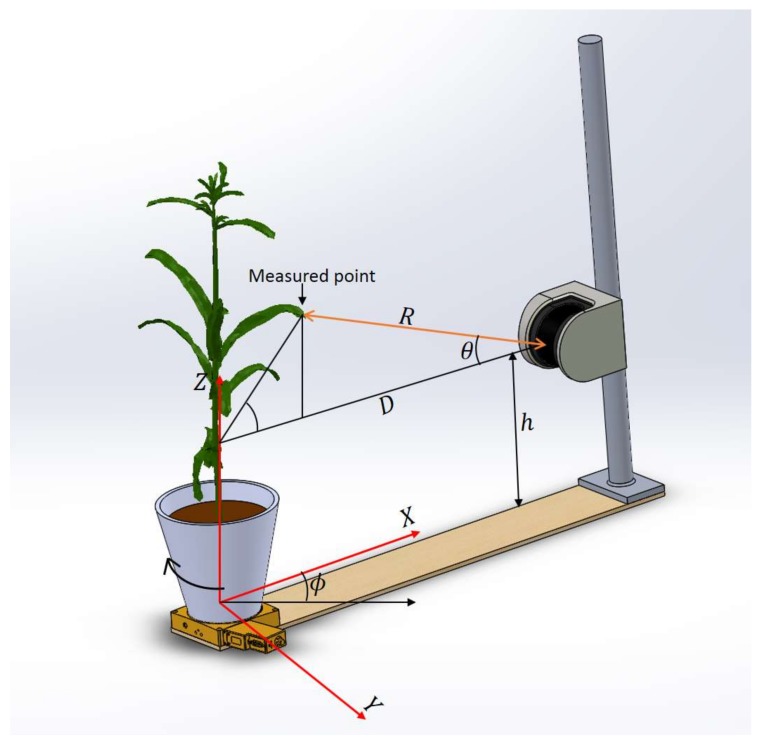
Coordinate system used to convert the range values from the LiDAR to Cartesian coordinates.

The vertical scanning plane of the LiDAR passing through the center of the rotation stage was considered the *XZ* plane with the center of the stage as the origin (Figure 2). The fixed distance D between the LiDAR and the center of the rotation stage was determined in advance by scanning a thin vertical rod placed at the center of the stage with the LiDAR. The measurements obtained from the LiDAR were converted into *XYZ* Cartesian coordinates using the following equations.

(1) X=(D−R ×cos θ)× cos ϕ,

(2) Y=(D−R ×cos θ)× sin ϕ,

(3) Z=h+R ×sin θ,

### 2.2. Point Cloud Processing Algorithm

**Figure 3:**
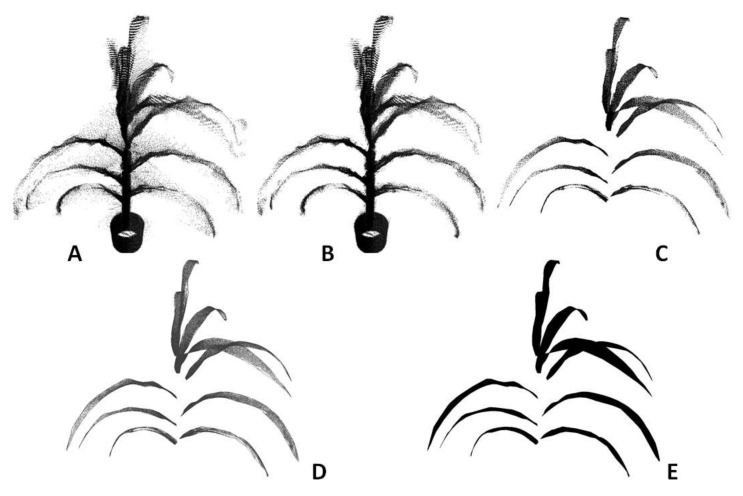
Example of 3D model of maize plant leaves after each step of point cloud processing: (**A**) raw point cloud of a maize plant derived by the instrument; (**B**) after voxelization and noise removal; (**C**) after clustering and segmentation; (**D**) after surface fitting and triangulation; and (**E**) a 3D rendering of the digital leaf surface model of the plant.

The algorithm to process the raw plant point cloud data includes these steps: background removal, voxelization, clustering and segmentation, triangulation and surface fitting, and plant morphological trait extraction. Figure 3 shows an example of a sorghum plant from its raw point cloud to the final 3D model by going through these processing steps. The computing time to process a typical plant is 60 s. 

Background removal: The point cloud data of plants generated by this new instrument contain part of the environment (e.g., the ceiling and walls of the room) and this part is called the background. The background is usually distinctive from the meaningful data of the plants in terms of the depth and is removed by limiting the *XYZ* range of the point cloud data.

Voxelization: Raw point cloud data are logged in a 2D matrix where every row corresponds to the 3D position (*XYZ*) of a point. The order of storing the positions is random and one point is not necessarily spatially close to its nearby points in the 2D matrix. This format is not optimal for data processing in later steps. To overcome this problem, voxelization was employed to transform the point cloud data into 3D matrix [26]. The resultant 3D matrix maintains the locality of the point cloud data, as every point is spatially close to its nearby points. Voxelization also helps to remove the noise in the point cloud further, because it estimates the point density of each voxel. Noise commonly has lower point density and therefore can be removed by setting a suitable threshold (which is determined heuristically).

Clustering and segmentation: The pot and the stem were removed from the data by filtering the points within a suitable radius from the center. This step made the leaves to be spatially separated from each other. K-means clustering was then used to segment the points that belonged to each leaf by feeding the k-value as the number of leaves in the plant [27].

Surface fitting and triangulation: Once the points belonging to a leaf were segmented, LOWESS (locally weighted scatterplot smoothing) was used to reconstruct the leaf surface [28]. At each local region of the cluster, a quadratic polynomial function was fitted to a subset of data points using weighted least squares. The process was repeated for all the segmented leaves in a plant.

**Figure 4:**
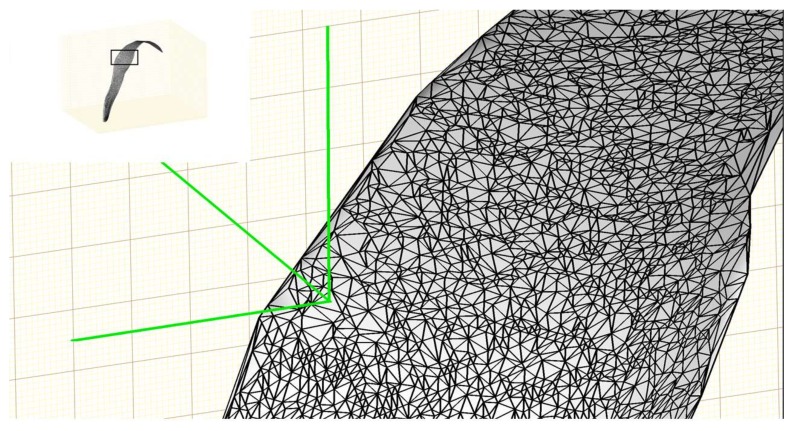
Example of triangulation of a fitted leaf surface in order to calculate leaf area, leaf inclination angle, and leaf angular distribution.

Although the surface can be well represented using some functions after surface fitting, it cannot be used directly to generate a 3D model. The surface needs to be discretized to a 3D triangular mesh before it can be visualized and processed further using computer graphics. The Delaunay triangulation algorithm [29] was used to generate a triangular mesh based on a set of points such that no point is inside the circumference of any triangle in the mesh. A triangular mesh was generated in the *XY*-plane using the *X* and *Y* values of the original 3D points and the *Z* value corresponding to each vertex of the triangle was obtained through the polynomial function of the fitted surface. The triangular mesh was elevated into the 3D space using the *Z* values and a 3D triangular mesh for a leaf was completed. The reconstructed 3D mesh of a plant leaf is shown in Figure 4.

Plant morphological traits extraction: Four plant morphological traits were extracted from the leaf surface model as shown in Figure 4—area of individual leaf, total leaf area of the plant, leaf inclination angle (the angle at which a leaf emerges with respect to the stem), and leaf angular distribution of each plant. Individual leaf area was obtained by adding the area of all the triangular meshes that form the leaf surface (Figure 4). Similarly, total leaf area was obtained by adding the area of all individual leaves from a plant. Leaf angle was measured as the angle between the leaf blade and the horizontal surface at each leaf node. To obtain leaf angular distribution, first the orientation (surface normal) of each triangle was obtained and the fraction of this triangle area to the total plant leaf area was calculated. The elevation angle in the surface normal vector was extracted (0° means horizontal leaf and 90° means vertical leaf) and the corresponding proportion was summarized into 10° bins. Finally, the histogram of area fractions versus the angle bins was plotted as leaf angular distribution.

### 2.3. Experiment to Validate the Instrument

To validate the instrument for leaf area and leaf angle measurement, 20 plants (ten maize (B73) and ten sorghum (Tx430)) were grown in the LemnaTec phenotyping greenhouse at the University of Nebraska-Lincoln. Plants were grown under non-stress conditions. The validation experiment began after the plants were six weeks old. In each week, four plants (two maize and two sorghum) were randomly selected for data collection. Therefore, data collection was completed in five weeks, in which the plants spanned several vegetative stages (e.g., for maize, from V6 to V11, plant height roughly 1.6 m at V11). This procedure allowed the instrument to be validated on plants of varying sizes and canopy complexity.

The data collection steps were as follows. First, the selected plant was scanned with the new instrument to obtain its raw 3D point cloud. Second, an RGB image of the plant was taken. The purpose of the RGB image was to (1) allow a side-by-side comparison of each plant and its point cloud and 3D model generated by the instrument; and (2) measure the angle between the plant’s individual leaves with respect to its stem as the reference method of leaf inclination angle. After imaging, all leaves on the plant were cut and leaf area was measured by a leaf area meter (Model LI-3100C, LI-COR Biosciences, Lincoln, NE, USA).

The raw plant 3D point cloud was processed with the algorithms described in Section 2.2 to obtain the area of individual leaves and the whole plant, inclination angle of individual leaves, and leaf angular distribution of the whole plant. MATLAB (version 2016, MathWork^®^, Natick, MA, USA) was used for point cloud processing. A correlation analysis was conducted to relate parameters derived from the 3D leaf surface model to those derived from the reference methods. Accuracy was assessed using R^2^ (coefficient of determination) and mean absolute error (MAE).

## 3. Results and Discussion

**Figure 5:**
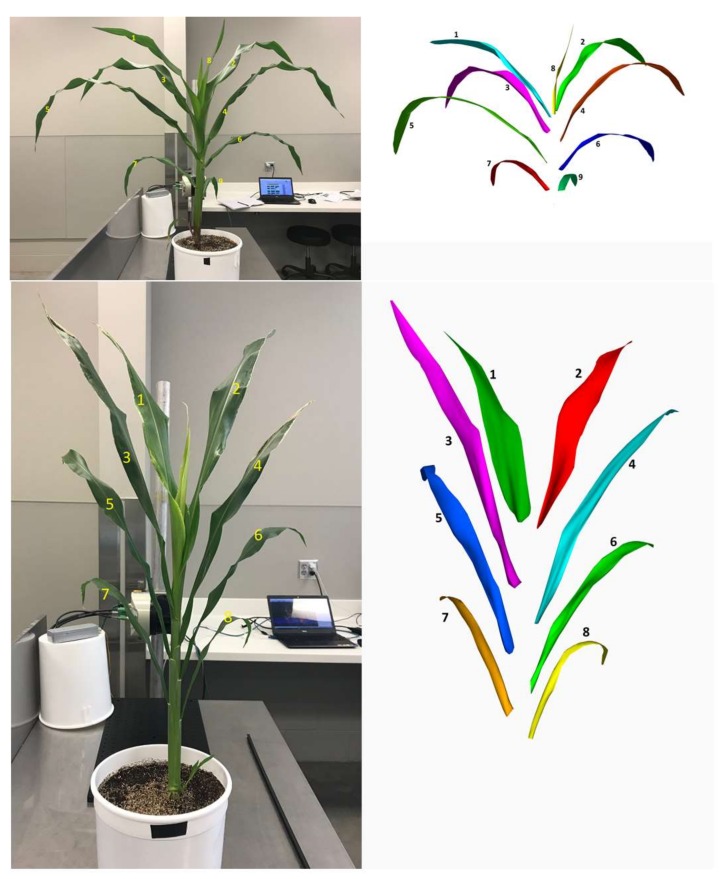
Side-by-side comparison of the RGB image of a maize and a sorghum plant and their 3D models obtained by the new instrument. The segmented individual leaves are labeled (1 through 9 for maize, and 1 through 8 for sorghum) sequentially for easy comparison.

Figure 5 shows the 3D model of a maize and sorghum plant after point cloud processing. It demonstrates that the 3D models were similar to the actual plants with regard to the overall canopy structure. The maize plant had nine leaves, all of which were reconstructed in 3D. Similarly, the sorghum plant had eight leaves; and they were reconstructed successfully in 3D as well.

**Figure 6:**
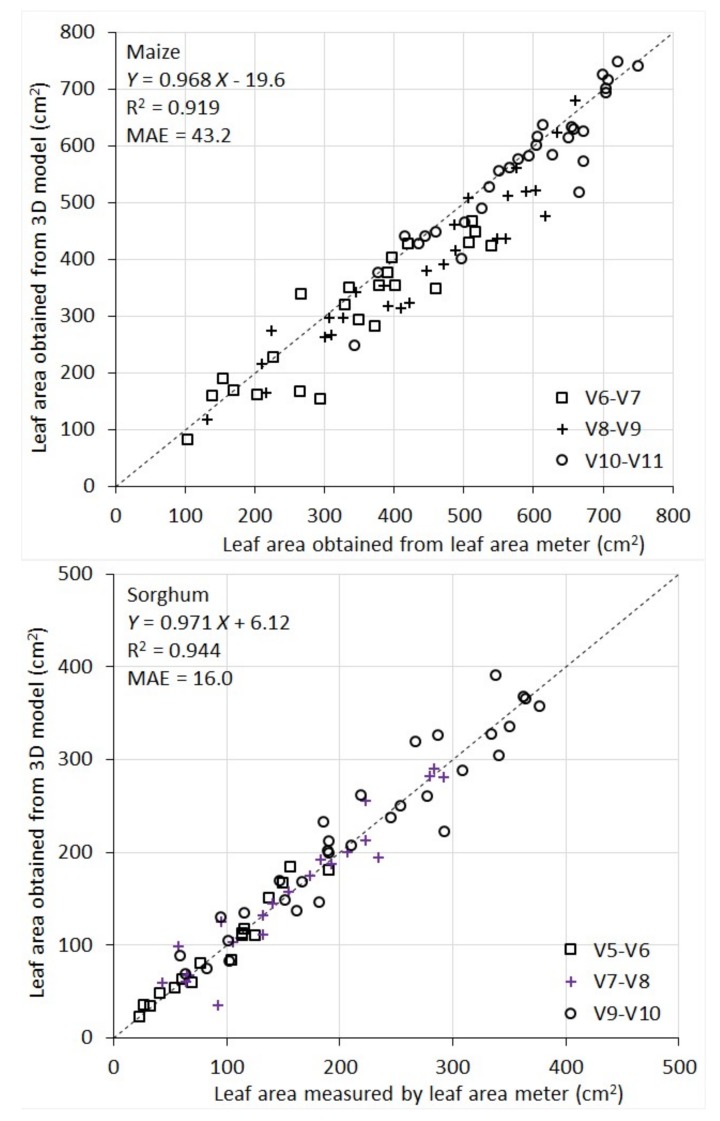
Scatterplot of individual leaf areas of 10 maize and 10 sorghum plants measured by the leaf area meter vs. obtained from 3D plant models. Different legends indicate different growth stages.

It can be seen from Figure 6 that 3D models give accurate, direct measurement of area of individual leaves. For maize, R^2^ between model-derived leaf area and the reference measurement was 0.92 and MAE was 43.2 cm^2^. Given the average maize leaf area of 463 cm^2^ in this study, the MAE represents an error rate of 9.3% of the average maize leaf area. For sorghum, R^2^ between the two sets of measurement was 0.94 and MAE was 16.0 cm^2^. The average sorghum leaf area is 170 cm^2^. Therefore, the MAE represents an error rate of 9.4% of the average.

The performance of the instrument for leaf area measurement is also comparable across the developmental stages being evaluated (for maize V6 to V11, and for sorghum V5 to V10). This can be seen in Figure 6, where the scatters of the points for the different stages are consistent with each other. 

For maize, there appears to be a systematic underestimation of individual leaf area by the 3D models compared to the area meter measurement. A closer examination of maize leaves (and their images) reveals that they have many local fine wrinkle structures particularly at leaf edges. The LiDAR point clouds are not quite effective to capture these local fine structures. Surface fitting (with 2nd order polynomials) and triangulation tend to smooth them into a flat surface, which leads to a systematic underestimate of the leaf area. On the contrary, sorghum leaves are smoother and therefore better represented by low-order polynomials in surface fitting and triangulation, which leads to lower systematic errors in leaf area modeling.

In addition to the systematic underestimate of leaf area in maize plants, there are also random errors in the leaf area measurement for both species by the instrument (some overestimate some underestimate). The random error components are attributed to three sources. Firstly, although the rotational stage largely addresses the occlusion problem, some occlusion still occurs for a subset of plants, in particular those with more complex structure. Partially occluded leaves cannot be reconstructed accurately (hence underestimated). Secondly, the LiDAR has an intrinsic accuracy limitation in distance ranging. This random error makes a smooth leaf surface appear rough during surface fitting and triangulation, thus leading to an overestimate of the area. Thirdly, the stem of the plants are thicker at the bottom and thinner at the top. Since we apply a constant distance filter to remove the stem points in point cloud processing, it causes slight area underestimate for top leaves and overestimate for bottom leaves.

**Figure 7:**
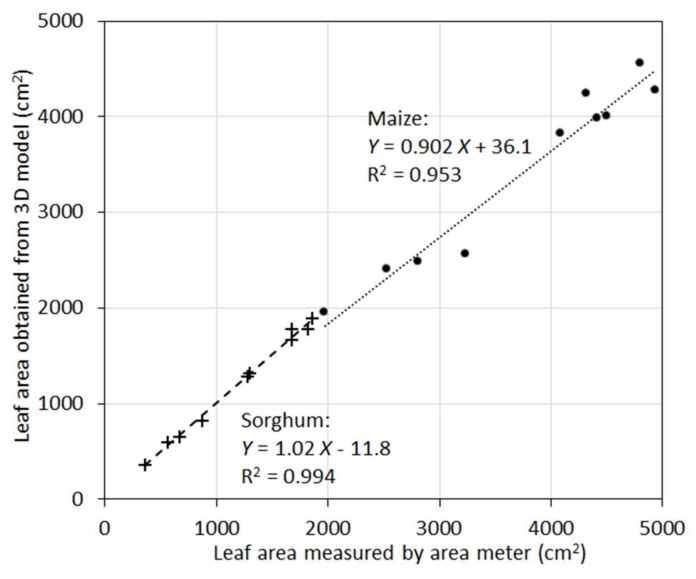
Scatterplot of total leaf areas of the 10 maize plants (circles) and 10 sorghum plants (crosses) measured by the leaf area meter vs. obtained from 3D plant models.

In many applications, it is the total leaf area rather than areas of individual leaves that needs to be measured or modeled. When individual leaf area was aggregated to total leaf area of a plant, the accuracy of the 3D models was increased (Figure 7). For maize, R^2^ was 0.95, and for sorghum R^2^ was 0.99. Aggregation from individual leaves to the whole plants removed random errors (overestimation vs. underestimation) and increased correlations. A closer examination indicated that the 10 maize plants were clustered into two groups in terms of total leaf area. This clearly inflated the R^2^ value as the maize plants exhibited larger scatter around the regression line compared to sorghum. Nevertheless, the high R^2^ values for both species suggest the new sensor can be a powerful tool to measure this important plant trait with higher accuracy.

In plant phenotyping, RGB images from multiple side views and top view were commonly used to estimate plant leaf area [5,12]. Because images only capture projected leaf area on 2D, empirical equations were developed to relate multi-view 2D projected leaf areas to the actual leaf area in 3D. The problem with this approach is that the empirical equations are dependent on plant species, their developmental stage, and even treatment effects. This represents a limitation of using RGB imaging to measure leaf area because, for each application, some plants have to be destructively sampled to build the empirical equation, which would lower the throughput of analysis. On the contrary, the platform we have developed directly measures plant leaf area in 3D. It eliminates the need of destructive sampling and will greatly improve the throughput of measurements.

**Figure 8:**
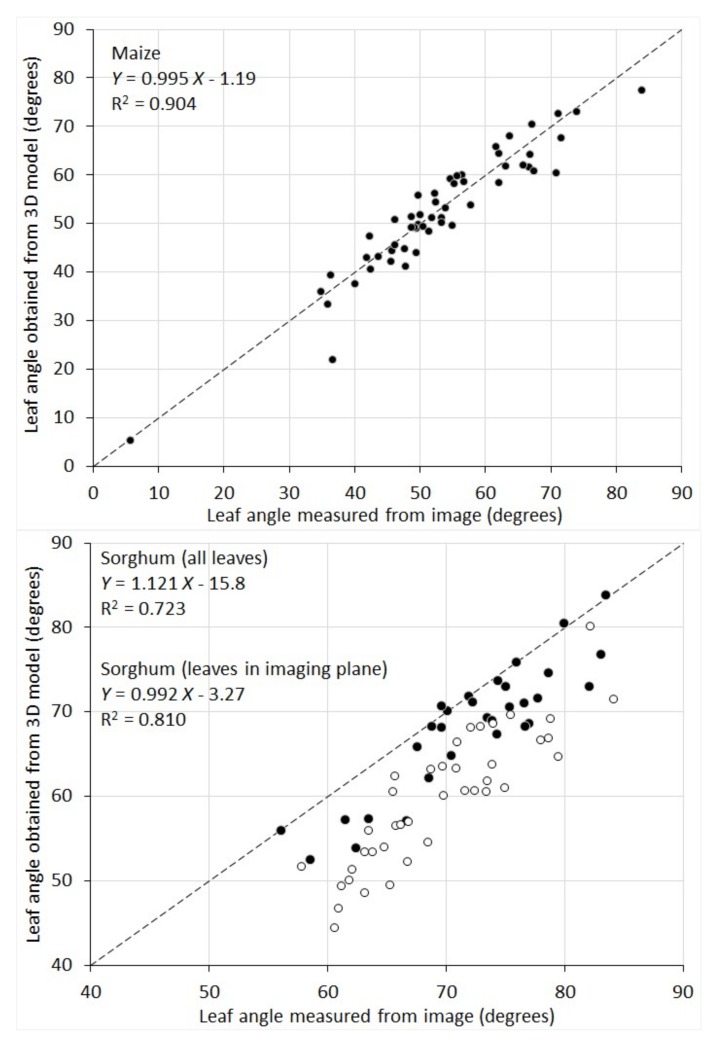
Scatterplot of individual leaf angle of ten maize and ten sorghum plants measured from their RGB images vs. obtained from 3D plant models. For sorghum, solid dots represent the leaves aligned in the imaging plane and hollow dots represent the leaves not in the imaging plane.

The correlation between leaf inclination angles measured from 2D images and those obtained from the 3D model was given in Figure 8. For maize plants, the two sets agree with each other well (R^2^ = 0.904) and the difference appears mainly to be random errors. Sorghum plants show a lower correlation (R^2^ = 0.723). There is an overall overestimation for leaf angle measured from the 2D images. This is because, for maize, there exists a plane in 3D where all the plant leaves are aligned. Since RGB images are taken in this plane, the images preserve the leaf inclination angle well. Whereas for sorghum plants, their leaves are distributed in all directions. Measurements of leaf inclination angle from their images lead to an overestimation for the leaves that grow into or out from the imaging plane. To make a better comparison, we selected the sorghum leaves that are aligned in the imaging plane by examining both the plant images and the 3D surface models. When only this subset of leaves is considered (black circles in the sorghum plot of Figure 8), the R^2^ value is 0.81 and the systematic underestimation is greatly reduced.

The 3D plant leaf models created by our instrument therefore overcome the limitation for leaf inclination angle retrieval from 2D images, and further enables the calculation of leaf angular distribution, which is even more challenging with 2D imaging.

**Figure 9:**
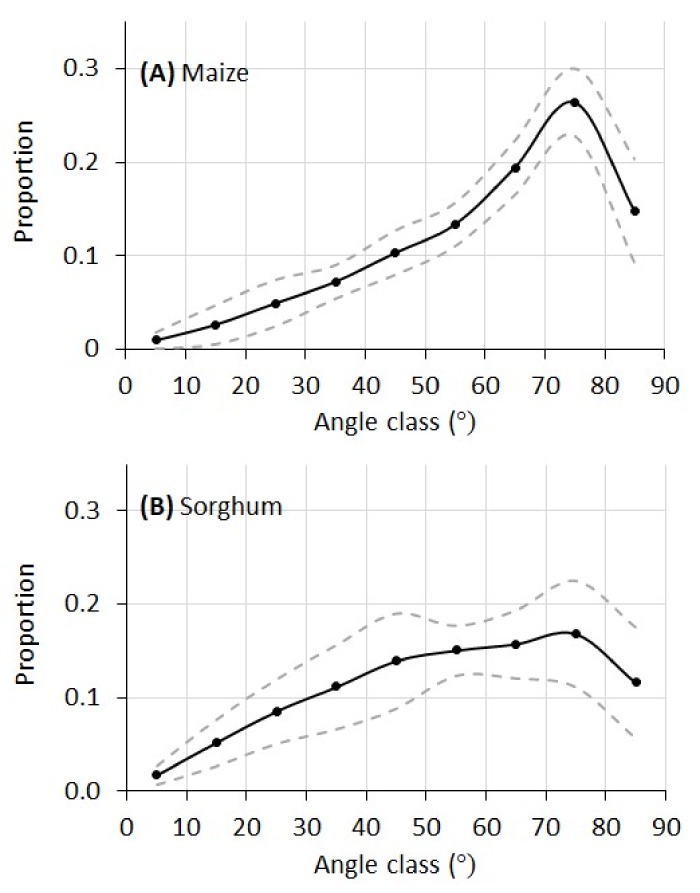
Leaf angular distribution of the ten maize plants and ten sorghum plants derived from their 3D leaf surface model. Solid line is the average proportion and the dashed lines are one standard deviation from the average.

The leaf angular distributions of the maize and sorghum plants in this experiment are given in Figure 9. For maize, the proportion of leaf area increased quickly from lower angles (horizontal leaf) to higher angles and peaked at 75°. For sorghum, the distribution of leaf angle increased more gradually from lower to higher angles. Whereas the maize plants have a single peak at 75°, the leaf angular distribution of the sorghum plants has a plateau between 45° and 75°.

As stated earlier, manual direct measurement of leaf angular distribution (LAD) needs alignment of an inclinometer at each and every leaf segment of a plant. For plants like maize and sorghum with large and curvy leaves, this could be very tedious if not impossible. Therefore, a validation of the LAD curves in Figure 9 with manual measurement was not conducted. However, limited published data on LAD provide validation on our measurements. For example, a maize canopy LAD reported in Ross [30] shared much similarity compared to our measurement in Figure 9A, with a rapid increase from low to high angles and a peak at 75°. A sorghum canopy LAD reported in Goel and Strebel [31] also matched our measurement in Figure 9B, with a gradual increase of leaf proportion from low to high angles with the largest proportion between 45 and 75°. Theoretically, plant canopy LADs can be modeled by a general ellipsoidal function with Campbell’s *x* parameter [11,32]. It can be seen that both maize and sorghum plants in this study exhibited typical erectophile distribution (meaning higher proportion of vertical leaf segments than horizontal leaf segments), which can also be verified from the RGB images of these plants. 

The leaf angle of both maize and sorghum plants was studied [33,34] to elucidate its genetic control. A very important aspect of leaf angle is that planting density can be higher for genotypes exhibiting more erect angular distribution. The new instrument can potentially be used as a nondestructive and rapid tool for selecting genotypes with desirable leaf angle and angular distribution for crop improvements, or evaluating large mapping or mutant populations to understand the genetic control of these leaf-angle related phenotypes [35].

Although the validation results demonstrated the potential of the new instrument, a few limitations are noted and should be worked on to improve the instrument. First, the maize and sorghum used to test the new instrument have a relatively simple plant structure and large leaves, compared to plants like soybean or cotton. The denser leaf structure of these species makes it more challenging to capture the complete point cloud of the canopy (due to occlusion), and smaller leaves further reduce the efficiency for surface reconstruction. Secondly, a few system parameters are provided manually, including starting and stop angles and LiDAR height for point cloud generation, as well as the threshold for point cloud noise removal and the leaf number for k-means clustering in point cloud processing. This is undesirable in practical settings. Lastly, if the stem of the plant is not upright, removing the stem part from the raw point clouds will be challenging.

We would also like to improve the instrument in the following two aspects. First, we will incorporate lasers of multiple wavelengths into the instrument, which would allow for the measurement of chemical or physiological traits of plants. Second, we will seek to integrate the instrument with an automated conveyor belt (such as LemnaTec Scanalyzer system [36,37]) to maximize its capacity for plant morphological trait measurement.

## 4. Conclusions

This paper reported the development and validation test of a new LiDAR-based instrument for high-throughput nondestructive measurement of plant morphological traits for single maize and sorghum plants. The following conclusions are drawn from this study.

- The instrument effectively generates a 3D point cloud of plants at a 360° view, with a measurement speed of 2 min per plant.
- The point cloud processing pipeline reduces point clouds to digital leaf surface model, with a processing speed of 60 s.
- Both the leaf area and the leaf inclination angle obtained from the 3D leaf surface model are highly correlated with those measured by the validation methods (R^2^ from 0.72 to 0.99).
- Generation of leaf angular distribution from 3D leaf surface model is demonstrated.
- With some future improvement, the instrument can potentially fill the current technological gap to enable direct, rapid, and nondestructive measurements of plant leaf area and leaf angular distribution.
